# Bivalves as Emerging Model Systems to Study the Mechanisms and Evolution of Sex Determination: A Genomic Point of View

**DOI:** 10.1093/gbe/evad181

**Published:** 2023-10-18

**Authors:** Filippo Nicolini, Fabrizio Ghiselli, Andrea Luchetti, Liliana Milani

**Affiliations:** Department of Biological, Geological and Environmental Science, University of Bologna, Bologna, Italy; Fano Marine Center, Fano, Italy; Department of Biological, Geological and Environmental Science, University of Bologna, Bologna, Italy; Department of Biological, Geological and Environmental Science, University of Bologna, Bologna, Italy; Department of Biological, Geological and Environmental Science, University of Bologna, Bologna, Italy

**Keywords:** *Dmrt* genes, DUI, molluscs, nonmodel organisms, sex-determining genes, phylogeny

## Abstract

Bivalves are a diverse group of molluscs that have recently attained a central role in plenty of biological research fields, thanks to their peculiar life history traits. Here, we propose that bivalves should be considered as emerging model systems also in sex-determination (SD) studies, since they would allow to investigate: 1) the transition between environmental and genetic SD, with respect to different reproductive backgrounds and sexual systems (from species with strict gonochorism to species with various forms of hermaphroditism); 2) the genomic evolution of sex chromosomes (SCs), considering that no heteromorphic SCs are currently known and that homomorphic SCs have been identified only in a few species of scallops; 3) the putative role of mitochondria at some level of the SD signaling pathway, in a mechanism that may resemble the cytoplasmatic male sterility of plants; 4) the evolutionary history of SD-related gene (SRG) families with respect to other animal groups. In particular, we think that this last topic may lay the foundations for expanding our understanding of bivalve SD, as our current knowledge is quite fragmented and limited to a few species. As a matter of fact, tracing the phylogenetic history and diversity of SRG families (such as the *Dmrt*, *Sox*, and *Fox* genes) would allow not only to perform more targeted functional experiments and genomic analyses, but also to foster the possibility of establishing a solid comparative framework.

**Significance**

In this perspective, we provide an examination of the phylogenetic diversity of *Dmrt* genes, a sex-determination (SD)-related gene family, to address the importance of bivalves in SD studies. By analyzing their taxonomic distribution and sequence diversity, we show how such a comparative study may set a common ground plan to settle down targeted functional experiments and essays. This kind of approach should be applied more extensively in future studies, especially when dealing with understudied organisms.

## Introduction

Bivalves are the second largest clade in molluscs, counting >18,000 species (https://www.catalogueoflife.org/, accessed December 16, 2022) distributed at all depths and in all marine environments, as well as in some freshwater habitats. Thanks to their high diversity and peculiar biological features, they have been proposed as promising model organisms for investigating a wide array of biological, ecological, and evolutionary issues, from mitochondrial biology and evolution to the physiological plasticity under fluctuating environmental conditions (Milani and Ghiselli 2020; Ghiselli et al. 2021). In this context, bivalves may serve as a compelling model system to investigate the evolution and characteristics of SD as well, thanks to the diversity of their reproductive modes and genomic features. Nonetheless, this research field has been largely overlooked and many aspects of bivalve reproductive biology remain uncharacterized. In this perspective, we address the topic by first examining the relevant questions that bivalves may help to answer regarding the processes and patterns of SD, and then providing a case study in the field of comparative genomics.

## Open Yet Inspiring Topics in Bivalve SD

Despite the socioeconomic and scientific importance of bivalves, the knowledge concerning the genetic and molecular bases of their SD system is quite limited, and its study has been mostly ignored. Yet, bivalves may constitute a novel model system in SD studies that are as intriguing and valuable as other well-established models such as vertebrates, insects, and plants (Tree of Sex Consortium 2014), as they may provide complementary perspectives in many aspects of SD evolutionary studies. Topics such as 1) the transition between environmental and genetic SD, 2) the evolution of sex chromosomes (SCs), 3) the mitonuclear interaction, and 4) the evolution of SD-related genes (SRGs), can largely benefit from the integration with bivalve studies. But many others are likely to emerge as research in the field progresses.

### Transitions Between Environmental and Genetic SD

**Fig. 1.**
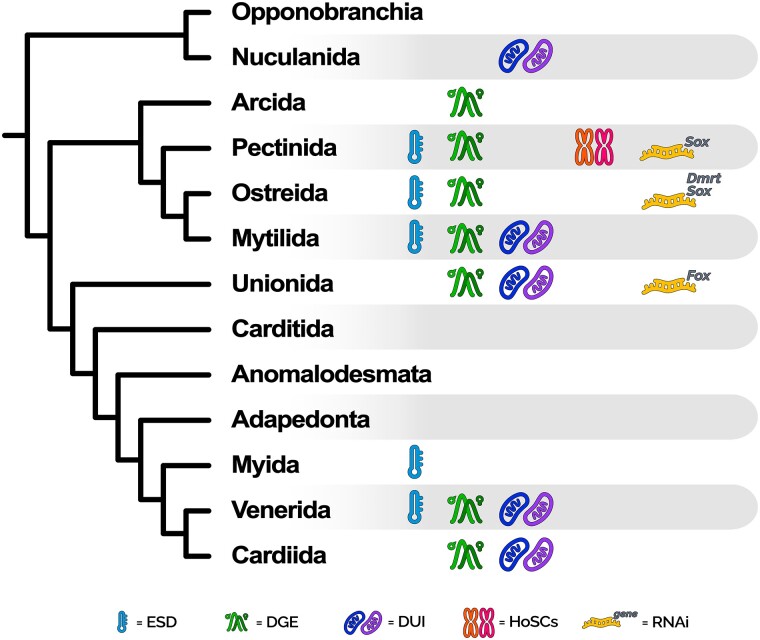
Graphical summary of the available knowledge and experiments concerning the genetic basis of SD in bivalves, at the level of major taxonomic orders (as reported in WoRMS; accessed on or before March 14, 2023). For each bivalve clade, the following is reported: 1) the availability of records of ESD; 2) the availability of differential gene expression (DGE) experiments specifically intended to investigate sex-biased or sex-specific genes; 3) whether the DUI of mitochondria has been reported in at least one species; 4) whether HoSCs have been identified in at least one species; 5) the availability of RNAi experiments for genes belonging to the *Dmrt*, *Sox*, and *Fox* gene families. The phylogenetic tree on the left has been drawn on the basis of the most widely accepted topology for bivalves, according to analyses based on nuclear markers and morphological data. The tips of the tree correspond to major bivalve orders, except for Opponobranchia and Anomalodesmata, which represent higher-level taxonomic ranks. References for the availability of data and experiments can be found throughout the main text.

Clues from several works seem to suggest that both genetic and environmental factors are involved in bivalve SD, thus implying that a mixed system may exist (reviewed in Breton et al. 2018). The traditional dichotomy between environmental SD (ESD) and genetic SD (GSD) seems inapplicable in most bivalve species, where ESD and GSD rather represent the two ends of a continuum of mixed and plastic conditions. A weak distinction between ESD and GSD is also found in amphibians, reptiles, and teleost fish, three clades in which environment-dependent SD has been largely studied. Here, the interaction—or even the transition—between the two sexual systems has been reported in many species, suggesting that sex-determining mechanisms can be extraordinarily plastic (Bachtrog et al. 2014; Capel 2017). Adding a representative and diverse group of Lophotrochozoa (Protostomia) to those vertebrate taxa can widely expand the comparative framework of the investigation, allowing us to better understand the evolution of SD as a whole. In bivalves, ESD has been studied mostly in oysters, where hermaphroditic species show an effect of temperature on SD (reviewed in Breton et al. 2018; fig. 1). Oysters may indeed constitute a prolific model to examine how the SD pathways are shaped in the presence of different initial triggers and highly dynamic reproductive backgrounds. In fact, various sexual systems can be found in oysters such as 1) a strictly gonochoric population, 2) the coexistence of simultaneous hermaphroditic with strictly gonochoric individuals in the same population, 3) the possibility of sex change according to environmental conditions, and 4) the presence of both parasitic dwarf males and free-living males in the same species (Collin 2013). Consequently, oysters may be extremely useful to understand how epigenetic control is involved in sex change, how gene regulatory networks can sustain the occurrence of different hermaphroditic conditions within gonochoric populations, and whether certain SD systems are more labile than others (Abbott 2011).

### Evolution of SCs

So far, heteromorphic SCs (i.e., SCs showing strong morphological differentiation; HeSCs) have never been observed in bivalves (Breton et al. 2018), while the first evidence of homomorphic SCs (i.e., SCs showing little or no differentiation; HoSCs) comes from a very recent study on several scallop species, where a nonhomologous origin of the SD system has been proposed for different subfamilies (fig. 1; Han et al. 2022). Theory predicts that, once originated, SCs will eventually turn into HeSCs because of the recombination arrest in the sex-determining region (Bachtrog et al. 2014; Beukeboom and Perrin 2014; Han et al. 2022). Nonetheless, HoSCs are much more widespread in the animal kingdom than expected, sometimes also being of ancient age (Bachtrog et al. 2014; Han et al. 2022).

Species from the order Pectinida may thus be useful to investigate what determines the long-term maintenance of HoSCs and which genomic architectures and molecular dynamics prevent HeSCs from evolving in bivalves. Additionally, they may be taken as model systems to investigate the origin of SCs in relation to the sexual systems and the route by which molecular pathways have been reprogrammed in the transition between different SD mechanisms (Han et al. 2022).

Researchers have been addressing this topic mainly in snakes, ratites, and sturgeons (Bachtrog et al. 2014; Han et al. 2022 and references therein), although scallops currently hold the oldest HoSC pairs, which date back to about 350 Myr. The system is thus of great importance to investigate the role of sex-biased gene expression and selection forces in the long-term stability of SCs (Han et al. 2022), as well as the intertwining between SD systems.

### Mitonuclear Interactions

An additional pivotal topic in bivalve biology, tentatively connected to SD, regards the doubly uniparental inheritance (DUI) of mitochondria, a process in which two highly divergent mitochondrial genomes are transmitted uniparentally through the maternal and paternal lineages, respectively, through eggs and sperm. This process, which has been reported in more than a hundred bivalve species from five different orders (fig. 1; Gusman et al. 2016; Capt et al. 2020), has been proposed to interact with the major nuclear pathways that primarily establish the sexual identity in a way that can resemble the cytoplasmatic male sterility (CMS) of plants (Ghiselli et al. 2013; Breton et al. 2022). In CMS, specific mitochondrial chimeric open reading frames (ORFs) cause the pollen to be sterile, while certain nuclear loci act in counterbalance to restore male fertility when occurring in the same individual. This Red-Queen scenario, in which balancing selection shapes the evolution of both CMS and restorer-of-fertility genes and keeps the two sexes viable, has also been hypothesized to be acting on bivalve DUI species (Ghiselli et al. 2013; Xu, Iannello, et al. 2022), where additional and effectively transcribed ORFs have been observed in both the male- and female-inherited mitochondrial lineages (Milani et al. 2013, 2014).

Clearly, if a functional interplay between DUI and SD in bivalves is proven, this will provide new research questions regarding not only bivalve biology itself but also broader evolutionary topics (e.g., are there any converging traits between DUI and CMS systems? What is the degree of plasticity of such mitochondria-related SD systems? Are mitochondria-related SD systems more widespread in eukaryotes than currently thought of?).

### Evolution of SRGs

Considering this intricate scenario of SD mechanisms and the wide diversity of bivalves, in the last few years, many differential transcription analyses have been performed on several species in an attempt to identify the most probable SRGs (fig. 1; e.g., Milani et al. 2013; Zhang et al. 2014; Chen et al. 2017; Capt et al. 2018; Shi et al. 2018). Interestingly, certain genes consistently emerged across different bivalve species as being substantially more transcribed in one sex (sex biased) or exclusively transcribed in one sex (sex specific), suggesting their potential involvement in the SD pathway. These genes mainly belong to the *Dmrt*, *Sox*, and *Fox* families, which play a role in various developmental processes (including the SD cascade) in most animals (Marshall Graves and Peichel 2010; Bachtrog et al. 2014; Beukeboom and Perrin 2014). Members of these three gene families are also included in the working model for the SD regulatory network proposed for the Pacific oyster *Crassostrea gigas* by Zhang et al. (2014), in which *CgSoxH* (which belongs to the *Sox* family) promotes male gonad development by activating *CgDsx* (which belongs to the *Dmrt* family) and inhibiting *CgFoxL2* (which belongs to the *Fox* family); *CgFoxL2*, when not inhibited by the pair *CgSoxH*/*CgDsx*, promotes female gonad development. Similarly, Han et al. (2022) appointed *FoxL2* as a putative SD gene in the two scallop species, *Patinopecten yessoensis* and *Chlamys farreri*.

If their pivotal role in SD of bivalves is confirmed, an evolutionary genomic analysis may help in better understanding why members of the above-mentioned gene families appear particularly prone to be recruited in the SD cascade also in distantly related species, as it is observed for *Dmrt1* and *Sox3* homologs in vertebrates (Marshall Graves and Peichel 2010; Bachtrog et al. 2014; and the following section). Furthermore, considering the occurrence of mixed SD systems in bivalves, *Dmrt*, *Sox*, and *Fox* genes may provide new perspectives on the influence of different environmental cues on the molecular evolution of animal SRGs. However, to date, experiments have been limited to molecular cloning, differential transcription, and tissue localization of such genes (Liang et al. 2019; Sun et al. 2022), while only a few have directly investigated their biological functions in bivalves, for example, through post-transcriptional silencing of target mRNAs (RNA interference [RNAi]; fig. 1; e.g., Liang et al. 2019; Wang et al. 2020; Sun et al. 2022).

Overall, *Dmrt*, *Sox*, and *Fox* genes are highly interesting targets to be investigated in the framework of bivalve SD and have indeed obtained much more attention than the study of SCs or the role of environmental cues. However, much work is still to be done in order to understand their function in the SD signaling pathway and their evolutionary history.

## The Case of the *Dmrt* Gene Family in Bivalves

Among the SRG candidates identified in bivalves, *Dmrt* genes (named after *doublesex* [*dsx*] from *Drosophilamelanogaster* and *male abnormal-3* [*mab-3*] from *Caenorhabditis elegans*) are of particular interest. As a matter of fact, in vertebrates, besides their role in placode neurogenesis and somite patterning (reviewed in Mawaribuchi et al. 2019), *Dmrt* genes are also involved in the development of male gonads and the maintenance of the testicular function (Sun et al. 2022). Their role in the specification and organization of male sexual characters seems indeed to be common across Metazoa, suggesting that a similar function may have already been present in the Bilateria common ancestor (Kopp 2012; Beukeboom and Perrin 2014).

**Fig. 2.**
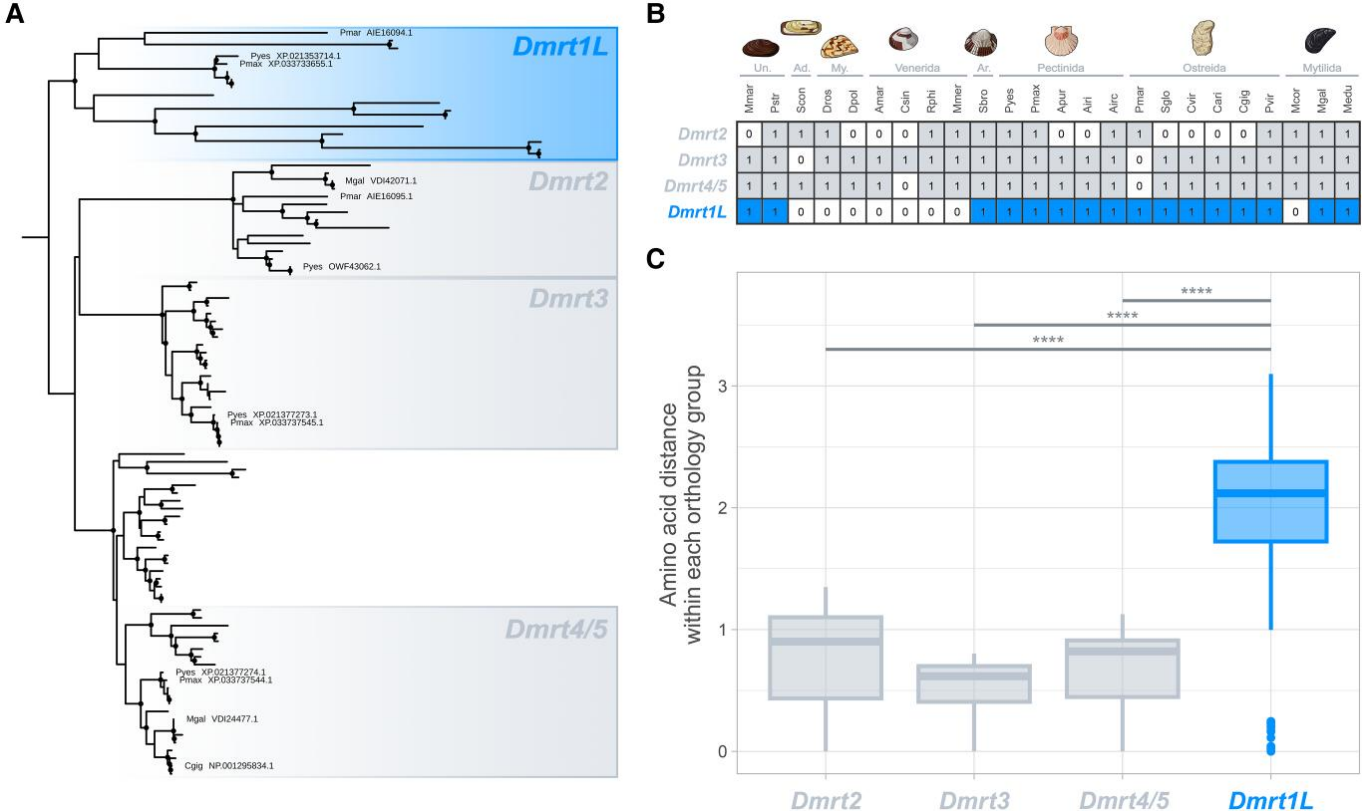
Phylogenetic tree (*A*) and taxonomic distribution (*B*) of *Dmrt* genes in bivalves, and a comparison of amino acid pairwise distances within *Dmrt1L* and the other *Dmrts* (*B*). (*A*) *Dmrt* orthologs from bivalve genome assemblies were obtained by using the HMMsearch (HMMER toolkit; Eddy 2011) with the Pfam HMM profile of the DM domain (PF00751). Amino acid alignment was obtained with MAFFT-DASH (Rozewicki et al. 2019), manually inspected to remove poorly aligning sequences, and trimmed with trimAl (gap threshold of 60%; Capella-Gutiérrez et al. 2009). The phylogenetic analysis was carried out using IQ-TREE 2 (Minh et al. 2020) with default parameters. Nodes with bootstrap values ≥85 are marked with filled black circles. The tree was rooted according to Evensen et al. (2022). *Dmrt* genes analyzed by Evensen et al. (2022) were used as references to annotate the various orthology groups, and accession numbers are reported in the tree. The phylogenetic tree with all annotated tips and nodes can be accessed on supplementary material online. (*B*) Taxonomic distribution of identified *Dmrt* genes in bivalve genomes. Orders as reported in WoRMS (accessed on or before March 14, 2023) and in figure 1 are specified. (*C*) Pairwise amino acid distances were computed for amino acid sequences within each *Dmrt* orthology group identified in the tree, with the R package “phangorn” (Schliep 2011) under the JTT substitution model. After checking for normality with the Shapiro–Wilk test (*W* = 0.88544, *P* < 2.2e−16) and for group effect with the Kruskal–Wallis test (*P* < 2.2e−16), the pairwise Wilcoxon rank-sum test was used to compare the distributions of pairwise amino acid distances of *Dmrt1L* and the other *Dmrts*. The horizontal bars mark the statistically significant results with *P* < 2.2e−16 (“****”) (Bonferroni correction for the multiple test was applied). The list of genome assemblies used for these analyses and species identifiers can be found in table 1. Un., Unionida; Ad., Adapedonta; My., Myida; Ar., Arcida.

The first attempts to dig inside the phylogenetic history and diversity of bivalve *Dmrt* genes have been provided by Li, Zhang, et al. (2018) and Evensen et al. (2022): besides retrieving all the canonical genes (i.e., *Dmrt2*, *Dmrt3*, and *Dmrt4/5*), their inferences brought to light a monophyletic *Dmrt* group (named *Dmrt1L*, which stands for *Dmrt1-like*) that appears to be private to molluscs and present in several bivalve species. The *Dmrt1L* monophyletic group is also confirmed when expanding the analysis by mining genomes from a wider range of bivalve taxa (table 1; fig. 2*A*), suggesting that *Dmrt1L* genes are widespread in bivalves and were likely present in their common ancestor (Evensen et al. 2022). In particular, *Dmrt1L* genes can be successfully retrieved in species of the orders Mytilida, Ostreida, Pectinida, Unionida, and from *Scapharca broughtonii* (Arcida), while the opposite holds for Venerida, *Sinonovacula constricta* (Adapedonta), and *Dreissena* spp. (Myida; fig. 2*B*). Clearly, the absence of *Dmrt1L* genes demands further investigations, as it may derive from errors in genome assembly and annotations.

**Table 1:** List of Bivalve Genomes From Which *Dmrt* Genes Have Been Extracted

Species	Species ID	Order	Assembly Level	BUSCO Score	Sequencing Method	Reference	NCBI Accession Number
*Anadara* (*Scapharca*) *broughtonii*	Sbro	Arcida	Chromosome	C: 91.2% [S: 85.6%, D: 5.6%], F: 2.6%, M: 6.2%	HiSeq X	Bai et al. (2019)	NA
*Sinonovacula constricta*	Scon	Adapedonta	Chromosome	C: 92.5% [S: 80.4%, D: 12.1%], F: 3.4%, M: 4.1%	Illumina HiSeq X; PacBio Sequel	Ran et al. (2019)	GCA_007844125.1
*Dreissena polymorpha*	Dpol	Myida	Chromosome	C: 86.9% [S: 75.1%, D: 11.8%], F: 6.4%, M: 6.7%	PacBio Sequel; Illumina HiSeq	McCartney et al. (2022)	GCA_020536995.1
*Dreissena rostriformis*	Dros	Myida	Scaffold	C: 75.2% [S: 73.2%, D: 2.0%], F: 15.2%, M: 9.6%	Illumina HiSeq	Calcino et al. (2019)	GCA_007657795.1
*Mytilus unguiculatus (coruscus)*	Mcor	Mytilida	Chromosome	C: 80.0% [S: 79.1%, D: 0.9%], F: 7.7%, M: 12.3%	Oxford Nanopore PromethION	Yang et al. (2021)	GCA_017311375.1
*Mytilus edulis*	Medu	Mytilida	Scaffold	C: 83.7% [S: 64.5%, D: 19.2%], F: 5.2%, M: 11.1%	Oxoford Nanopore; Illumina HiSeq X	Corrochano-Fraile et al. (2022)	GCA_905397895.1
*Mytilus galloprovincialis*	Mgal	Mytilida	Scaffold	C: 80.3% [S: 47.5%, D: 32.8%], F: 8.8%, M: 10.9%	Illumina HiSeq2000	Gerdol et al. (2020)	GCA_900618805.1
*Perna viridis*	Pvir	Mytilida	Scaffold	C: 99.4% [S: 99.0%, D: 0.4%], F: 0.2%, M: 0.4%	Illumina HiSeq2500	Inoue et al. (2021)	GCA_018327765.1
*Magallana (Crassostrea) ariakensis*	Cari	Ostreida	Chromosome	C: 94.6% [S: 90.9%, D: 3.7%], F: 0.9%, M: 4.5%	Oxford Nanopore PromethION	Li et al. (2021)	GCA_020567875.1
*Magallana (Crassostrea) gigas*	Cgig	Ostreida	Chromosome	C: 98.5% [S: 67.6%, D: 30.9%], F: 0.3%, M: 1.2%	PacBio Sequel	Peñaloza et al. (2021)	GCF_902806645.1
*Crassostrea virginica*	Cvir	Ostreida	Chromosome	C: 98.1% [S: 58.6%, D: 39.5%], F: 0.3%, M: 1.6%	PacBio_RSII	Gómez-Chiarri et al. (2015)	GCF_002022765.2
*Saccostrea glomerata*	Sglo	Ostreida	Scaffold	C: 88.9% [S: 85.3%, D: 3.6%], F: 5.1%, M: 6.0%	Illumina HiSeq	Powell et al. (2018)	GCA_003671525.1
*Argopecten irradians concentricus*	Airc	Pectinida	Scaffold	C: 94.8% [S: 93.9%, D: 0.9%], F: 3.7%, M: 1.5%	Illumina HiSeq	Liu et al. (2020)	GCA_004382765.1
*Argopecten irradians irradians*	Airi	Pectinida	Scaffold	C: 94.8% [S: 93.9%, D: 0.9%], F: 3.7%, M: 1.5%	Illumina HiSeq	Liu et al. (2020)	GCA_004382745.1
*Argopecten purpuratus*	Apur	Pectinida	Scaffold	C: 89.2% [S: 88.5%, D: 0.7%], F: 5.0%, M: 5.8%	PacBio Sequel	Li, Liu, et al. (2018)	NA
*Pecten maximus*	Pmax	Pectinida	Chromosome	C: 98.5% [S: 74.7%, D: 23.8%], F: 0.4%, M: 1.1%	PacBio, Chromium 10X, Hi-C	Kenny et al. (2020)	GCF_902652985.1
*Mizuhopecten* (*Patinopecten*) *yessoensis*	Pyes	Pectinida	Scaffold	C: 98.6% [S: 75.2%, D: 23.4%], F: 0.4%, M: 1.0%	Illumina HiSeq	Wang et al. (2017)	GCF_002113885.1
*Margaritifera margaritifera*	Mmar	Unionida	Scaffold	C: 92.6% [S: 82.3%, D: 10.3%], F: 3.2%, M: 4.2%	Illumina NovaSeq	Gomes-dos-Santos et al. (2021)	GCA_015947965.1
*Potamilus streckersoni*	Pstr	Unionida	Scaffold	C: 74.7% [S: 73.8%, D: 0.9%], F: 7.0%, M: 18.3%	PacBio Sequel; 10X Genomics	Smith (2021)	GCA_016746295.1
*Calyptogena (Archivesica) marissinica*	Amar	Venerida	Chromosome	C: 82.0% [S: 80.0%, D: 2.0%], F: 6.1%, M: 11.9%	PacBio Sequel; Illumina NovaSeq	Ip et al. (2021)	GCA_014843695.1
*Cyclina sinensis*	Csin	Venerida	Scaffold	C: 94.0% [S: 83.8%, D: 10.2%], F: 1.9%, M: 4.1%	Illumina HiSeq; PacBio	Wei et al. (2020)	GCA_012932295.1
*Mercenaria mercenaria*	Mmer	Venerida	Chromosome	C: 95.4% [S: 70.9%, D: 24.5%], F: 0.5%, M: 4.1%	PacBio; Illumina	Song et al. (2021)	GCF_014805675.1
*Ruditapes philippinarum*	Rphi	Venerida	Chromosome	C: 83.4% [S: 74.5%, D: 8.9%], F: 8.8%, M: 7.8%	Illumina HiSeq	Xu, Martelossi, et al. (2022)	GCA_026571515.1

Note.—For each species, the accepted name and the most-common synonym (in parentheses) are reported as found in WoRMS. NCBI accession numbers are provided, when available, as well as BUSCO scores of the predicted proteomes against the metazoa_odb10 data set (Manni et al. 2021).

The present analysis also supports a higher amino acid sequence divergence of the *Dmrt1L* orthology group with respect to the other *Dmrt* orthology groups (fig. 2*C*), which may be explained by a higher rate of sequence evolution related to their sex-biased expression in certain species (Zhang et al. 2014; Shi et al. 2015; Li, Zhang, et al. 2018; Evensen et al. 2022). This is consistent with what has already been observed for the SRGs *Dmrt1* and *dsx* in vertebrates and *Drosophila*, respectively (e.g., Bewick et al. 2011; Baral et al. 2019). In fact, sex-biased genes (including SRGs) often tend to evolve faster than unbiased genes at the level of protein sequences, when considering either male-biased genes (reviewed in Parsch and Ellegren 2013; Grath and Parsch 2016) or female-biased genes (e.g., Papa et al. 2017; Ghiselli et al. 2018). Another possible explanation for the higher amino acid divergence of *Dmrt1L* genes may lie in their expression breadth; that is, genes with a narrow tissue-specific expression tend to evolve faster than more ubiquitous genes (Parsch and Ellegren 2013; Xu, Martelossi, et al. 2022). As a matter of fact, *Dmrt1L* genes have been found to be significantly more transcribed in the gonadic tissue (particularly in testes) in *P. yessoensis* (Li, Zhang, et al. 2018) and *Cr. gigas* (Yue et al. 2021).

Understanding the role and molecular interactions of *Dmrt1L* genes in bivalve SD and gonad development would greatly enhance the possibility of outlining the evolutionary causes and consequences of their high amino acid divergence (fig. 2*C*), for example by linking the molecular evolution to the degree of pleiotropy. However, most of our knowledge on *Dmrt1L* biology is currently limited to the temporal and tissue localization of transcripts in a few species of bivalves (e.g., Li, Zhang, et al. 2018; Yue et al. 2021). In fact—apart from the work by Sun et al. (2022), who confirmed the role of *Dmrt1L* in the gonad development of *C. gigas* through noninvasive RNAi and found that the knocked down phenotype results in a size reduction of male gonads—no other experiments intended to elucidate the function of *Dmrt1L* genes in bivalves have been carried out so far (fig. 1). This clearly hinders any possible integration between molecular data and functional assays.

If the role of *Dmrt1L* as a major SD is confirmed, bivalves will become an intriguing clade to investigate why, in Metazoa, certain genes (namely, the *Dmrt* gene family) appear particularly prone to being recruited at the top of the SD cascade. To date, this phenomenon has been widely examined in vertebrates, where *Dmrt1* genes have independently gained a primary role in male SD in fish, amphibians, and birds, and are considered candidate sex-determining genes also in monotreme mammals (Marshall Graves and Peichel 2010; Beukeboom and Perrin 2014; Mawaribuchi et al. 2019). Bivalves may provide an alternative evolutionary scenario to study the selective forces and molecular modifications that support *Dmrt* genes in repeatedly taking over the SD process. In fact, since *Dmrt1L* genes seem to be restricted to molluscs (fig. 2*A*), it would be intriguing to clarify whether the putative involvement in the SD cascade of extant bivalve species is the result of shared ancestry or convergent evolution, which would establish a study system for the evolution of *Dmrt* genes parallel to that of vertebrates (see Capel 2017).

Obviously, *Dmrt1L* should not be expected to be the sole sex-determining gene. In fact, *FoxL2* has already been appointed as the female sex-determining gene in *P. yessoensis* and *Ch. farreri* (Han et al. 2022). Consequently, we should expect that other primary genetic determinants exist, consistent with the extremely high species diversity of the clade. Thus, bivalves may additionally serve as a valuable model system to study how genes from different families take over the SD cascade and are shaped by selection.

## Conclusions: Bivalves as New Models in the Study of SD

SD is undoubtedly a fascinating biological and evolutionary topic as much as it is challenging to investigate. Our understanding of the causes and consequences of the SD mechanism diversity strongly relies on the study of different systems and nonmodel model organisms (Bachtrog et al. 2014; Milani and Ghiselli 2020), which provide the foundation for depicting a comprehensive evolutionary and comparative framework in which new and coherent research perspectives can be grounded.

In recent years, bivalves have been gaining growing importance in many fields of biology, ranging from ecology to genomics, and from environmental biomonitoring to mitochondrial studies (Milani and Ghiselli 2020; Ghiselli et al. 2021), but they can be a valuable model to also address SD studies. The diversity of their life history traits provides indeed a challenging, yet extremely fascinating framework, to put the SD processes into an evolutionary context.

Bivalves can help us explain how ESD and GSD interplay with each other in response to environmental conditions, as a mixed system of both has been proposed to act in the establishment of bivalve sexual identity (reviewed in Breton et al. 2018). Moreover, the occurrence of the many existing variants of hermaphroditism and gonochorism even in closely related species, or within the same population, strongly suggests that the basic SD pathway (whether genetic, environmental, or mixed) should be plastic enough to sustain the existence of individuals of both sexes, thus providing the opportunity to study how SD gene regulatory networks are shaped and selected throughout evolution and how epigenetic regulation may influence SD. The unique DUI system further poses an undeniable challenge in SD studies, since it may represent an SD-linked mechanism that relies on the non-nuclear portion of the genome and may unfold many new research paths (Milani and Ghiselli 2020; Ghiselli et al. 2021). Nonetheless, much of the research effort on bivalve SD has been devolved to specific groups of socioeconomic importance such as Mytilida, Ostreida, Pectinida, and Unionida, while the other lineages of the bivalve phylogeny have been ignored (fig. 1). Our understanding of the SD processes of bivalves is thus restricted and mainly lacks a broad comparative framework in which to draw comprehensive evolutionary inferences.

Genes from the *Dmrt*, *Sox*, and *Fox* families, which are also involved in SD in other Metazoa, may be considered excellent genomic targets to study the processes and patterns of molecular evolution in sex-biased genes, as well as the recurrent recruitment of genes in the SD cascade. Also, identifying the major genetic regulators of SD in bivalves would burst the functional study of the interaction between ESD and GSD, by providing genetic targets that can be manipulated through RNAi and/or genome editing techniques to understand the role of environmental cues in SD. In the same way, knowing the main genetic actors of SD would allow researchers to identify SCs not only on the basis of in silico techniques (such as k-mer based or SNP methods) but also by less-expensive wet laboratory protocols (such as fluorescence in situ hybridization on metaphase chromosome plates). Furthermore, it would help researchers to understand whether and how the mitochondrial additional ORFs of DUI species interact with the SD system, by performing thorough gene expression analyses.

In conclusion, we strongly urge researchers to invest more resources in the integrative study of bivalve SD to unravel the many underlying mechanisms and expand our understanding of this biological process. Given our limited knowledge in the field, one of the first routes that should be undertaken may rely on the comparative study of SRGs of bivalves from a genomic perspective, because such type of data is nowadays growing at a rate faster than ever. Establishing such a genomic ground plan for understudied organisms will, in fact, allow researchers to develop evolutionary-aware experiments with better-selected genetic targets.

## Data Availability

Data analyzed in this perspective and R scripts used to generate plots can be accessed in supplementary material online deposited at the following GitHub repository: https://github.com/filonico/bivalve_sex_perspective.
